# Interplay of Agency and Ownership: The Intentional Binding and Rubber Hand Illusion Paradigm Combined

**DOI:** 10.1371/journal.pone.0111967

**Published:** 2014-11-04

**Authors:** Niclas Braun, Jeremy D. Thorne, Helmut Hildebrandt, Stefan Debener

**Affiliations:** 1 Department of Psychology, Neuropsychology Lab, Carl von Ossietzky Universität Oldenburg, Oldenburg, Germany; 2 Neurology Department, Klinikum Bremen Ost, Bremen, Germany; 3 Research Center Neurosensory Science, Carl von Ossietzky Universität Oldenburg, Oldenburg, Germany; 4 Cluster of Excellence “Hearing4all”, Carl von Ossietzky Universität Oldenburg, Oldenburg, Germany; University G. d'Annunzio, Italy

## Abstract

The sense of agency (SoA) refers to the phenomenal experience of initiating and controlling an action, whereas the sense of ownership (SoO) describes the feeling of myness an agent experiences towards his or her own body parts. SoA has been investigated with intentional binding paradigms, and the sense of ownership (SoO) with the rubber-hand illusion (RHI). We investigated the relationship between SoA and SoO by incorporating intentional binding into the RHI. Explicit and implicit measures of agency (SoA-questionnaire, intentional binding) and ownership (SoO-questionnaire, proprioceptive drift) were used. Artificial hand position (congruent/incongruent) and mode of agent (self-agent/other-agent) were systematically varied. Reported SoO varied mainly with position (higher in congruent conditions), but also with agent (higher in self-agent conditions). Reported SoA was modulated by agent (higher in self-agent conditions), and moderately by position (higher in congruent conditions). Implicit and explicit agency measures were not significantly correlated. Finally, intentional binding tended to be stronger in self-generated than observed voluntary actions. Results provide further evidence for a partial double dissociation between SoA and SoO, empirically distinct agency levels, and moderate intentional binding differences between self-generated and observed voluntary actions.

## Introduction

We usually take it for granted that our bodies are spatially extended in the world and that we are agents, acting upon the world. The sense of agency (SoA) refers to the phenomenal experience of initiating and controlling an action in order to bring about a change in the world [1], [2]: for instance voluntarily lifting one's arm (the motor aspect of an action) in order to pick up a glass (the intentional aspect of an action) [3]. As such, the SoA can be distinguished from the sense of ownership (SoO), which describes the feeling of myness or ownership an agent experiences towards his or her own body parts [4]. For voluntary actions (e.g. an agent voluntarily lifts his or her arm) the SoA and SoO naturally coincide, but both experiences can also be made in isolation. If someone else lifts an agent's arm, the agent still experiences a SoO for the arm but not a SoA. Hence, while under normal conditions a SoA is experienced only for voluntary actions, a SoO can exist for both voluntary actions and passive sensory experience [2]. It may also be that a perfectly adapted prosthetic device requires a SoO and SoA for the prosthesis. A better understanding of how these experiences relate to each other seems important in basic research and may also govern progress in different fields of neurorehabilitation.

Investigations of the SoO are often carried out using the rubber hand illusion (RHI) [5]. In the standard RHI paradigm, an artificial hand is placed visibly and in an anatomically plausible position in front of a participant, while the participant's own hand becomes hidden from view. The experimenter then repeatedly strokes both the artificial hand and the real hand in synchrony. In the majority of participants this results in a strong illusory SoO over the artificial hand and to a subjective mislocalization of the real hand's position towards the artificial hand, which is called proprioceptive drift. Interestingly, the illusion diminishes when the artificial hand is placed in misalignment to the real hand [6] or when the visual and tactile stroking is done asynchronously [5]. Moreover, it has been shown that an illusory SoO and proprioceptive drift can also be induced over a virtual 3D projection of a hand (the so called “virtual hand illusion”; VHI) and that here the strength of the illusion also depends on visuotactile synchrony [7], [8] and on different anatomical plausibility factors [9], [10].

Studies on the SoA are frequently based on variants of the intentional binding paradigm (see [11] for a review). The intentional-binding effect refers to the subjective compression of time experienced between a voluntary action and its external sensory consequences (see e.g., Moore & Obhi, 2012, for a review). A popular variant of this paradigm is the interval estimation approach. Participants report the perceived time interval between an action, such as a button press, and its subsequent sensory effect, such as a tone. A typical observation is that the time interval is only underestimated when the action is voluntary, but not when it is involuntary [1], [12]–[14]. Whereas one study found intentional binding only for self-generated voluntary actions and not for observed voluntary actions [15], others have reported that intentional binding occurs regardless of whether an action is self-generated or only observed, as long as the act is interpreted as voluntary and a biological agent clearly identifiable [16]–[18]. Moreover, whereas some studies have clearly revealed stronger binding effects for self-generated than observed voluntary actions [15], [19], others reported no difference in the strength of intentional binding between self-generated and observed voluntary actions, although both action types showed higher binding compared to a neutral no agent condition [16]–[18]. In contrast to this latter finding, some studies have also reported binding effects in the absence of an identifiable biological agent, suggesting that causal interference in general rather than voluntary action is the central explanatory factor of binding [20]–[22]. At present it is therefore not well understood to what extent self-generated and observed voluntary actions share similar binding mechanisms (i.e. how self-specific intentional binding actually is) and how much binding reflects causal inference. Moreover, we are not aware of any peer-reviewed study that has experimentally tested for the relevance of embodiment for intentional binding.

Although originally conceived as a unitary concept, recent theoretical and empirical works have argued for distinct SoA levels [1], [23]. For instance, Synofzik et al. [23] proposed a multifactorial two-step account, in which an implicit SoA level might be distinguished from an explicit SoA level. The implicit SoA level is a lower-level, pre-reflective and non-conceptual “feeling of agency”. Its phenomenology seems rather “thin”, only allowing for a rudimentary self-other distinction and not for an explicit attribution of who has caused an action, and it has been assessed by measures of intentional binding [1]. An internal prediction model has been suggested to contribute in this level of agency [15], [23]–[26], according to which every voluntary movement induces an efference copy or corollary discharge. If efference copy and actual sensory input match, a movement is perceived as self-generated and a feeling of agency arises. In the case of a mismatch between efference copy and sensory input, no feeling of agency occurs. The explicit SoA level in contrast reflects a higher-order, belief-like process that refers to a person's interpretation of being the agent of an action [23]. The explicit SoA level enables an explicit attribution of an action to an agent to be made as well as a reflection about who has caused an action. It is for instance assessed through retrospective agency evaluations that require participants to indicate their perceived level of contribution to an action [27]. The interplay of these two different SoA levels has however only been reported in one study so far [1].

Whereas the SoA has often been investigated with intentional binding paradigms, SoO studies frequently capitalize on the RHI or, more recently, VHI. Only a few studies have investigated both concepts concurrently [28]–[32]. Several studies by the Sanchez-Vives Group have used an active virtual hand that either moved in synchrony or moved in asynchrony with the participant's own movements and have thereby shown that visuomotor synchrony is sufficient in order to induce an illusory SoO and proprioceptive drift over a virtual hand, even in the absence of tactile stimulation [8], [29]. However, despite the virtual hand's movability in these studies, no explicit assessment of the SoA was conducted in these studies. Another, more recent, report investigated the SoA with the RHI paradigm. Kalckert and Ehrsson [28] used an active version of the RHI where the artificial hand was placed on an upper plate and the participant's real hand was placed directly below it, on a lower plate. The artificial hand's index finger was connected to the real hand's index finger by a tiny rod that went through the upper plate. As a result, whenever the participant moved the index finger up or down in a self-agent condition, or the rod was moved up or down by the experimenter, constituting an other-agent condition, the artificial hand's index finger moved correspondingly. Using this paradigm, Kalckert and Ehrsson [28] found a partial double dissociation between the SoA and SoO. Whereas an incongruent positioning of the artificial hand eliminated the SoO and did not affect the SoA, observed movements of the artificial hand diminished the participants SoA but left their SoO for the artificial hand intact. In this study, however, no implicit agency measure was used and the action scenario consisted only of autotelic rhythmic finger tapping, so any further intentional aspects (e.g. an external sensory event that results from the finger movement) could not be addressed.

By incorporating the intentional binding paradigm into the RHI paradigm, the general goal of the present study was to further investigate the complex interplay between SoA and SoO on subjective and behavioral levels. Specifically, our first aim was to replicate the findings of Kalckert and Ehrsson [28] by inducing a SoA and SoO for an artificial, moving hand, however this time with a more complex action task than autotelic finger tapping. Secondly, we asked whether intentional binding can be provoked by an artificial hand, and if so, how self-specific this action-effect binding would be and whether it would depend on embodiment. To this end, self-agent, other-agent and no agent conditions were realized for two artificial hand positions which were congruent or incongruent to the real arm position. Regarding the agent factor, it was hypothesized that self-generated and observed voluntary actions would show strong intentional binding when compared to a neutral control condition [16]–[18]. Moreover, based on the two aforementioned studies [15], [19], a (small) difference between the two action types was also predicted. Due to the lack of empirical evidence, no specific hypothesis was formulated regarding the role of hand position. Finally, a third research question investigated how the implicit and explicit SoA levels related to each other, and how the relationship between the two SoA levels was affected by the agent and position factors. The associations between intentional binding and proprioceptive drift, between the SoO and proprioceptive drift and between intentional binding and the SoO were also analyzed.

## Methods

### Participants

Twenty-eight right-handed participants (age range: 20–30) were recruited for the study. All participants were female, since no male artificial hand model was available. Participants were required to have normal or correct-to-normal-vision, no known history of psychiatric or neurological disorders, and were not taking any psychoactive medication. All participants gave written informed consent and were paid for their participation. None of them had previously participated in an intentional binding or RHI experiment and all participants were naive to the purpose of the study. The experiment was conducted in accordance with the current version of the Declaration of Helsinki and approved by the local ethics committee of the University of Oldenburg (University Oldenburg: Kommission für Forschungsfolgenabschätzung und Ethik). Three participants were excluded from statistical analyses, two due to technical reasons and one for failing to follow instructions.

### Apparatus

**Figure 1:**
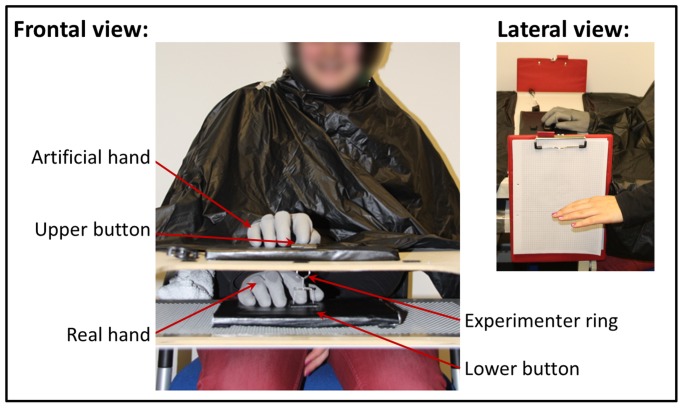
Experimental design, frontal view. Participants placed their right hand on the lower plate, whereas the artificial hand was placed directly above on the upper plate. The artificial hand's index finger and the lower button were connected via a string. Hence, whenever either the lower button was pressed by the participant (self-agency) or the ring was moved up or down by the experimenter (other-agency), the index finger of the artificial hand moved up or down accordingly. Lateral view. Illustrated is the measurement of the proprioceptive drift. Participants closed their eyes, stretched out their left arm and indicated with their left index finger the perceived height of their right hand. The height of the participant's left index finger was then marked by the experimenter onto the scale paper attached to each side of the table.

The experimental set-up is depicted in Fig. 1. Participants sat in front of a rectangular table (50×60 cm) consisting of a tabletop and a lower shelf. The vertical distance between the tabletop and lower shelf was 7.5 cm. One button press device was placed in the middle of the tabletop and another one directly below on the lower plate. The upper button was connected to a notebook so that button-presses could be recorded. Presentation software (version 14.9; Neurobehavioral Systems Inc., Albany, USA) was used for stimulus presentation. A life-sized plaster cast of a female human hand (18 cm in length, from the tip of the middle finger to the end of the wrist) was covered with a thin-gauge garden glove and served as our artificial hand. The hand was placed in the middle of the table top, parallel to the short sides. The artificial index finger was equipped with a rebounding joint and fixed to the upper response button, such that index finger movements could be realistically mimicked. A small string was attached to the lower side of the artificial finger's tip (invisibly covered under the glove) and was threaded through a hole in the tabletop to the area below, where it was split into two strands. The two strands were connected to the outer edges of the lower button, such that the participant's index finger could be comfortably placed on the lower key without touching the string. Attached to the string above the lower key was a small ring used by the experimenter in the other-agent conditions to move the string up and down. Hence, whenever either the lower button or the ring was moved up or down, the index finger of the artificial hand moved up or down accordingly.

Participants were instructed to wear a garden glove identical to the glove on the artificial hand and to place their right hand onto the lower shelf with the index finger placed on the lower button. Depending on the experimental condition (details in the next section), participants sat either on the long table side where the artificial hand was in alignment with their own right hand (congruent conditions) or on the long table side where the artificial hand was rotated through 180°, thus was in disalignment to their own right hand (incongruent conditions). In the congruent conditions, participants were instructed to place themselves in such a way that it appeared plausible for them that the artificial hand could be part of their body. In the incongruent conditions, participants were instructed to place themselves in a way that it appeared plausible for them that the artificial hand could be part of their body, if it were rotated by 180°. Throughout the experiment, a blanket was placed over the participant's shoulders and arm so as to cover the space between the artificial hand and the participant's body thereby facilitating the impression that the artificial hand was the participant's own outstretched hand (see figure 1). The experimenter's arm was also hidden under a cover so that the participant could not see when the experimenter was pulling the ring to synchronously move the artificial index finger and the participant's real index finger. Participants were instructed to leave their index finger on the lower button throughout the conduction of each condition. The lower button did not have any lifting force of its own, and only moved upwards when the string was also moved upwards. This arrangement ensured that the visual feedback was identical between self-generated and experimenter-generated button presses. In addition, this setup enabled near identical proprioceptive and tactile feedback for the two experimental conditions. It could thereby be assured that potential intentional binding differences would be attributable not to perceptual differences, but rather to the action type.

### Design and procedure

A 2×2 factorial design was used. The two factors of the main experiment were position (congruent vs. incongruent) of the artificial hand relative to the participant's perspective, and agent (self-agent vs. other-agent) relative to the participant's perspective. The four experimental conditions were therefore (1.) congruent–self-agent: the artificial hand was in alignment with the participant's hand and finger movements were executed by the participant; (2.) congruent–other-agent: the artificial hand was in alignment with the participant's hand, but the experimenter was executing the finger movements; (3.) incongruent–self-agent: the artificial hand was not in alignment with the participant's own hand (rotation by 180°), but the participant was executing the finger movements; (4.) incongruent–other-agent: the artificial hand was not in alignment to the participant's hand and the experimenter was executing the finger movements. The main experiment consisted of four blocks, one for each of the four experimental conditions. To avoid order effects, block order was counterbalanced across participants using a Latin square approach.

All four blocks were identically structured in the following way. First, an intentional binding phase was implemented, and this was followed by a free pressing phase. Afterwards proprioceptive drift was measured, and finally a questionnaire was used to assess the participant's experience of SoA and SoO. Throughout the intentional binding and free pressing phases, participants were instructed to look at the artificial hand and focus on the moving artificial index finger. The experimenter sat opposite the participant with his right hand covered under the blanket. In the incongruent conditions, the experimenter effected the index finger movements by moving the ring up and down (see figure 1). After each block, a break of approximately two minutes was included. During this break, participants relaxed before the experimenter gave instructions for the next upcoming block and the table was, where necessary, rotated by 180°. Raw data files and preprocessing scripts were made available in Dataset S1.

### Intentional binding

As in previous studies [1], intentional binding was used for the assessment of the implicit SoA level. Participants were instructed to estimate the time interval in ms between the onset of a button press and a subsequently played sound. Time interval estimations had to be verbally given to the experimenter. Whereas in the two self-agent conditions the participants effected the artificial index finger movements themselves, in the two other-agent conditions they experienced visually, by touch and proprioception, how the artificial hand's index finger moved without their voluntary contribution. Participants performed 45 trials in each of the four conditions. Following previous studies [12], [33], beep sounds were randomly presented via a headphone either 100 ms (15 trials), 400 ms (15 trials) or 700 ms (15 trials) after the onset of each button press. Participants were told that beep tones would occur at random within the range of 0 to 1000 ms. In order to take sufficient time for RHI induction into account, the first 15 trials of each intentional binding phase were excluded from the statistical analysis. This figure was derived from our piloting studies that suggested that, despite some individual differences, most participants had perceived the illusion by this point. Each participant performed a practice run and a control condition before the experiment. In the practice run, 15 trials were presented, in which participants had to estimate the time interval between two sounds and received feedback after each trial. This was done to acquaint the participants with the estimation of small time intervals and reduce effects of time misestimation unrelated to the intentional binding effect. The same latencies that were used in the main experiment were also used for the practice run. A control condition was adopted from Pooninan and Cunnington [16] and served as a no-agent condition, in that no agent was clearly identifiable. This condition was introduced in order to detect potential systematic effects of time misestimation unrelated to the intentional binding itself. The no-agent condition consisted of 45 trials (as in the four main experimental conditions) in which participants had to estimate the time interval between two sounds (as in the practice run, but without any feedback). Intentional binding was defined as the average underestimation of the actual time interval in percentage ([actual value–estimated value]/actual value) across all 45 trials.

### Free pressing phase

For comparability reasons, free pressing phases were also included into our experiment, as in Kalckert's and Ehrsson's study [28] During these phases, participants either moved the artificial hand's finger up and down in a 1 Hz semi-regular rhythm themselves (self-agent conditions) or they “experienced” how the artificial hand's index finger moved up and down in this rhythm but without their contribution (other-agent conditions). The free pressing phases lasted one minute.

### Proprioceptive drifts

As in previous studies [28], [34]–[36], proprioceptive drift was used as an implicit measure of limb ownership. After each free pressing phase, participants were instructed to close their eyes, stretch out their left arm and indicate with their left index finger the perceived height of their right hand. The height of the participant's left index finger was then marked by the experimenter onto a board with scale paper attached to each side of the table (see figure 1). Proprioceptive drift was defined as the amount of shift in centimeters from the real hand towards the artificial hand. Positive values indicated an upward drift towards the artificial hand, negative values a downward drift away from the real hand.

### Questionnaire data

A 12-item questionnaire adopted from Kalckert and Ehrsson [28] was used for the explicit assessment of the SoA and SoO (table 1). The questions were read by the experimenter at the end of each block and the participants indicated their level of agreement on a 7-point Likert scale, ranging from −3 (“totally disagree”) to +3 (“totally agree”). Four statements referred to the SoA (e.g. “The artificial hand moved just like I wanted it to, as if it were obeying my will.”) and four statements referred to the SoO (e.g. “I felt like the artificial hand was part of my body.”). The remaining four statements served as control statements. Two of these related to the SoA (e.g. “I felt as if the artificial hand was controlling my will”) and two to the SoO (e.g. “It felt as if I no longer had a right hand, as if my right hand had disappeared.”). The control statements included illusion-related statements that did not capture the phenomenal experience of agency or ownership. Hence, with successful SoA induction, the SoA-related questions should have high affirmative ratings in the two self-agent conditions, and low or negative ratings in the SoA control questions, as responses to these questions should not specifically be affected by the agency manipulation. Likewise, with successful SoO induction, the SoO related questions should have high affirmative ratings in the two congruent conditions, whereas the ratings for the SoO-control questions should be low or negative. All questions appeared in a pseudo-randomized order. Questionnaire evaluation followed Kalckert and Ehrsson (2012). The four SoA statements were averaged to obtain a single value for the perceived SoA level and the four SoO statements to obtain a single value for the perceived SoO level. Moreover, the SoO control scores and SoA control scores were calculated by averaging the two control statements for agency and ownership. As in former studies [6], [28], [37], the illusion criterion was set to > = +1. Hence, an average score > = +1 was interpreted on the group level, as participants having affirmed the statement (i.e. they had experienced a SoA or SoO).

**Table 1:** Questionnaire for the SoA and SoO.

Category	Statement	Order of questions
SoO-judgment	I felt like I was looking at my own hand.	3
	I felt like the artificial hand was part of my body.	6
	It seemed as if I were sensing the movement of my finger in the location where the artificial finger moved.	8
	I felt as if the rubber hand were my hand.	10
SoA-judgment	The artificial hand moved just like I wanted it to, as if it were obeying my will.	1
	Whenever I moved my finger, I expected the artificial finger to move in the same way.	4
	I felt as if I were causing the movement that I saw.	7
	I felt as if I were controlling the movements of the artificial hand.	11
SoO-control:	It appeared as if the artificial hand were drifting towards my real hand.	5
	It felt as if I no longer had a right hand, as if my right hand disappeared.	2
SoA-control:	I felt as if the artificial hand were controlling my will.	9
	It seemed as if the rubber hand had a will on its own.	12

### Statistical analysis

The current experiment included four main experimental dependent variables (perceived SoA level, perceived SoO level, proprioceptive drift and intentional binding) and three control variables (SoO control scores, SoA control scores and the no agent control condition for intentional binding). Prior to data analyses, all variables were checked for normal distribution using the Kolmogorov-Smirnov test. Where appropriate, parametric statistics were used. The main experiment consisted of a 2×2 factorial design (see section 2.3). Hence, for each main dependent variable, two-way repeated measures ANOVAs with the two factors position (congruent vs. incongruent) and agent (self-agent vs. other-agent) were conducted.

For the explicit assessment of the SoA and SoO, control questions were available. This allowed us to test how specific the experimental manipulations were, whether for example, they would only affect the illusion-specific questions or those questions that went beyond the mere phenomenal experience of agency or ownership. In order to statistically validate this, pairwise comparisons of the perceived SoA levels and the SoA-control scores were conducted in the two self-agent conditions by means of t-tests. Likewise, the perceived SoO levels were compared to the SoO control scores in the two congruent conditions, using a t-test for the congruent–other-agent condition and a Wilcoxon signed-ranked test for the congruent–self-agent condition.

In order to evaluate binding in the absence of an explicit agent, t-tests between each experimental condition and the no-agent condition were conducted. Moreover, in order to study the relationships between the outcomes of the questionnaire-based evaluation of the SoA and SoO, the intentional binding measure and the proprioceptive drift, we calculated Pearson correlation coefficients for each possible combination of these four variables. Correlation coefficients were calculated for each of the four experimental conditions separately and also as a single value for all conditions combined. The combined correlation coefficients were obtained by calculating the mean value for each variable for each participant and then calculating the correlation coefficient of these mean values.

## Results

### Perceived SoA level

**Figure 2:**
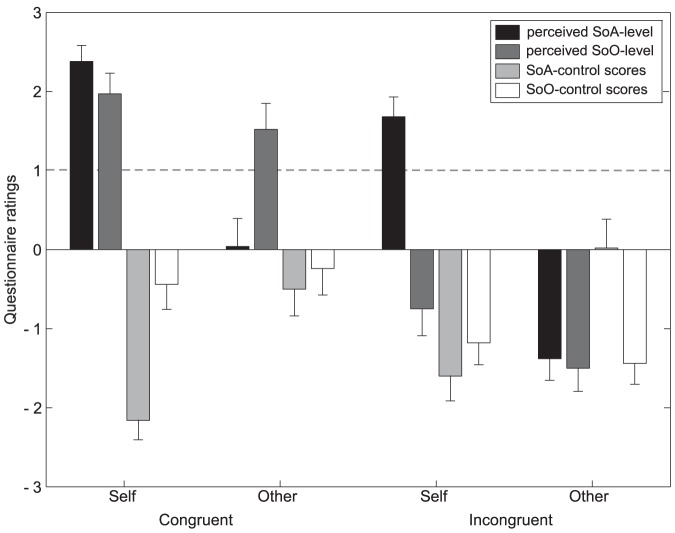
Explicit assessment of the SoA and SoO. Mean (+SEM) questionnaire ratings for different experimental conditions are shown. Values above a criterion of 1, illustrated by a dashed line, reflect report of the illusion (cf. REF). Congruent/incongruent refers to positioning of the artificial hand relative to the participants hand, self/other refer to the mode of agency. SoA = sense of agency; SoO = sense of ownership.

The perceived SoO and SoA levels are depicted in figure 2. The designated illusion criterion (+1) was met for the perceived SoA level in the congruent self-agent condition (M = 2.38; SD = 1.01) and incongruent self-agent condition (M = 1.68; 1.25), but not for the perceived SoA level in the incongruent other-agent condition (M = −1.38; SD = 1.36) and congruent other-agent condition (M = 0.04; SD = 1.76) and also not in any conditions for the SoO control score (all below 0.1). Planned comparisons between the perceived SoA levels and SoA control scores were significant both for the congruent self-agent condition (T(24) = 7.88; p<.001) and for the incongruent self-agent condition (Z = −4.38; p<.001), confirming the illusion-specificity of the experimental manipulations. A 2×2 repeated measures ANOVA revealed a significant main effect for the factor agent (F(1,24) = 60.54; p<.001) reflecting that the perceived SoA level was higher in the two self-agent conditions as compared to the two other-agent conditions. This finding reflects the expected occurrence of a SoA in these two conditions. Moreover, the ANOVA revealed a main effect for the factor position (F(1,24) = 15.09; p = .001), confirming that the perceived SoA level was higher in the two congruent conditions compared to the two incongruent conditions. The interaction between agent and position factors was significant as well (F(1,24) = 4,67; p = .041). Post hoc t-tests were significant for all pairwise comparisons, that is, congruent self-agent vs. congruent other-agent (T(24) = 5.68; p<.001), congruent self-agent vs. incongruent self-agent (T(24) = 2.71; p = .012), congruent self-agent vs. incongruent other-agent (T(24) = 11,44; p<.001), congruent other-agent vs. incongruent self-agent (T(24) = −3.09; p = .005), congruent other-agent vs. incongruent other-agent (T(24) = 3.82; p = .001) and incongruent self-agent vs. incongruent–other-agent (T(24) = 8.84; p<.001).

### Perceived SoO level

The designated illusion criterion was met for the perceived SoO level in the congruent self-agent (M = 1.97; SD = 1.31) and congruent other-agent condition (M = 1.52; SD = 1.64) but not for the perceived SoO level in the incongruent–self-agent (M = −0.75; SD = 1.69) and incongruent–other-agent condition (M = −1.50; SD = 1.46) and also not in any condition of the SoO control score (all below zero). Planned comparisons between the reported SoO-levels and SoO control scores were significant both for the congruent self-agent condition (T(24) = 6.02; p<.001) and for the congruent other-agent condition (T(24) = .373; p<.001), confirming the illusion-specificity of the experimental manipulations.

A 2×2 repeated measures ANOVA revealed a main effect of position (F(1,24) = 95.76; p<.001) in that a higher perceived SoO level was evident in the congruent compared to the incongruent conditions. The questionnaire results thus confirmed the predicted occurrence of a SoO in the relevant conditions. Moreover, a significant main effect for the factor agent (F(1,24) = 5.54; p = .027) was found, showing that the perceived SoO level was higher in the two self-agent conditions than in the two other-agent conditions. The interaction between agent and position factors was not significant (F(1,24) = 0.43; p = .517)).

### Proprioceptive drifts

**Figure 3:**
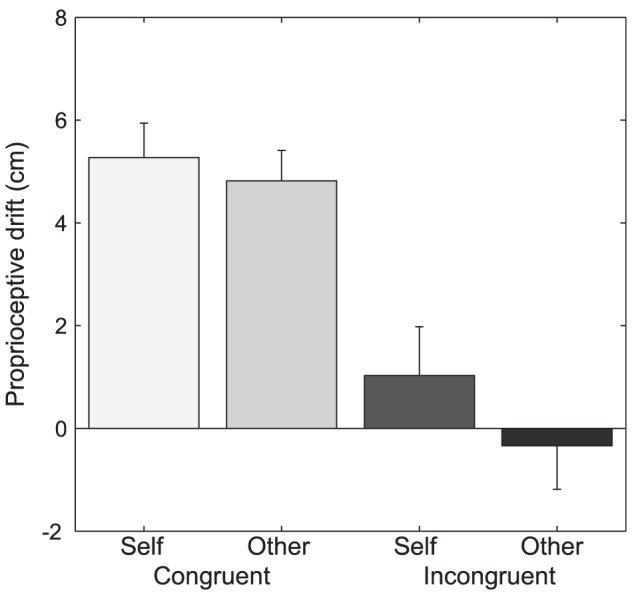
Proprioceptive drift. Values show mean (+SEM) proprioceptive drift in centimeters. Congruent/incongruent refers to positioning of the artificial hand relative to the participants hand, self/other refer to the mode of agency.

The proprioceptive drift results are illustrated in figure 3. A 2×2 ANOVA revealed a main effect of position (F(1,24) = 38.44; p<.001) in that the proprioceptive drift was on average larger for the two congruent compared to the two incongruent conditions. There was no main effect of agent (F(1,24) = 3.24; p = .084) and no interaction between the factors (F(1,24) = 1.16; p = .291). On average, the proprioceptive drift were most pronounced for the congruent self-agent condition (M = 5.27; SD = 3.33) and congruent other-agent condition (M = 4.81; SD = 2.96), whereas only a weak drift was found for the incongruent self-agent condition (M = 1.02; SD = 4.76) and no drift for the incongruent-other condition (M = −0,34; SD = 4.22).

### Intentional binding

**Figure 4:**
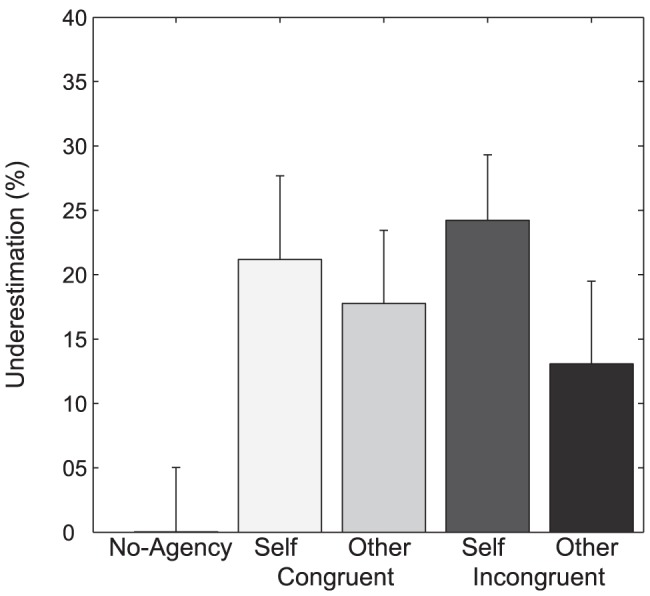
Intentional binding. Values show mean (+SEM) underestimation of time intervals in percentage. Congruent/incongruent refers to positioning of the artificial hand relative to the participants hand, self/other refer to the mode of agency.

Figure 4 illustrates the intentional binding results. In all of the four main experimental conditions, participants underestimated the actual time intervals between button presses and subsequent sounds by at least 13%. As predicted, no underestimation occurred for the “no-agent” control condition. Here, the time interval estimation was, on average, very accurate. Planned pairwise comparisons showed that in three out of the four main experimental conditions the time interval estimations were significantly shorter than in the neutral control condition. Significant differences were found for congruent self-agent vs. neutral (T(24) = 2.05; p = .025), congruent other-agent vs. neutral (T(24) = 2.04; p = .026) and incongruent self-agent vs. neutral (T(24) = 2.83; p = .004) comparisons, whereas no significant effect occurred for the incongruent other-agent vs. neutral (T(24) = 1.25; p = .110) comparison. A 2×2 repeated measures ANOVA revealed a trend for the main effect of agent (F(1,24) = 4.03; p = .056) in that the two self-agent conditions were stronger compared to the two corresponding other-agent conditions. There was no main effect for position (F(1,24) = 0.01; p = .892) and no significant interaction (F(1,24) = 0.04; p = .325). Hence, whereas being an agent versus observing an agent (self-agent vs. other-agent) was reflected in the intensity of intentional binding, the position of the hand did not affect intentional binding.

### Relationships between the measures

Additional analyses explored the associations between the main variables; table 2 summarizes the corresponding results. Regarding a possible relationship between perceived SoA-levels and perceived SoO-levels, strong and significant correlations were found for the congruent self-agent (r = .642; p = .001), congruent other-agent (r = .594; p = .002) and incongruent other-agent (r = .616; p = .001) conditions. A marginal correlation was found for the incongruent self-agent condition (r = .386; p = .057). Also the all-condition correlation revealed a strong association between the two explicit measures (r = .625; p = .001). The correlation coefficients between intentional binding and proprioceptive drift were marginally positive in the congruent self-agent (r = .354; p = .082), congruent other-agent conditions (r = .334; p = .103) and marginally negative in the incongruent self-agent condition (r = −.343; p = .093). In the incongruent other-agent condition no association was found (see table 2). A significant relationship between the two SoO measures (proprioceptive drift, perceived SoO-level) could not be confirmed by the correlation analysis. Associations were very weak and non-significant in three out of the four conditions and on the total level. Only in the incongruent other-agent condition a marginal correlation between the two measures was found (r = .341; p = .095). With respect to the relationship between the implicit (intentional binding) and explicit (perceived SoA level) SoA measures, the correlation coefficients were very low in three of the four conditions and did not reach significance. Again, only in the incongruent other-agent condition a marginal correlation emerged (r = .386; p = .057). No indication for significant associations between the perceived SoO level and intentional binding was found.

**Table 2:** Correlations between different SoA and SoO measures.

Pair of correlation		Blocks				
		Congruent self-agent	Congruent other-agent	Incongruent self-agent	Incongruent other-agent	total
Perceived SoA level vs. Perceived SoO level	r	.*642*	.594	.386	.*616*	.625
	p	*.001*	.002	.057	*.001*	.001
Perceived SoO level vs. Proprioceptive Drift	r	*.052*	*−.067*	*.003*	*.341*	.066
	p	*.804*	*−749*	*.987*	*.095*	.752
Perceived SoA level vs. Intentional binding	r	−.260	.072	−.070	.386	.188
	p	.210	.733	.739	.057	.367
Perceived SoO level vs. Intentional binding	r	.027	.068	−.011	.006	.115
	p	.897	.748	.960	.977	.586
Intentional Binding vs. Proprioceptive Drift	r	.354	.334	−.343	.007	.201
	p	.082	.103	.093	.975	.334

Cells with italic font indicate correlations testing for specific hypotheses. After correction for multiple comparisons, a critical p-value of p = .0083 (.05/6) was used.

## Discussion

The aim of this study was to further investigate the complex interplay between SoA and SoO by combining the intentional binding paradigm with the RHI-paradigm. Replicating Kalckert and Ehrsson [28] a SoO was clearly observed in our study, since the perceived SoO level was high in congruent and low in incongruent conditions. This finding confirms that an illusory SoO can not only be induced by an inanimate, artificial hand but also by a moving artificial hand [28], and that tactile stimulation is not necessary to induce this illusion [29]. Importantly, a continual updating of proprioceptive information resulting from the participant's moving index finger did not destroy the proprioceptive drift and illusory feeling of ownership. This suggests that even in the presence of constant postural changes, no recalibration of the proprioceptive system towards the participant's real hand position takes place, as long as the intermodal matching between vision and proprioception can be maintained. Moreover, the perceived SoA level was high in the two self-agent conditions but low in the two other-agent conditions. Taking both findings together, we thus confirm the double dissociation between the SoA and SoO, as previously reported by Kalckert and Ehrsson [28]. Incongruent positioning of the artificial hand eliminated the SoO but did not destroy the SoA, whereas observed movements of the artificial hand reduced the SoA but left the SoO for the artificial hand intact.

Importantly, the dissociation between the SoA and SoO was partial and incomplete. As was the case in the study of Kalckert and Ehrsson [28], the perceived SoO level was also modulated by the agent factor and the perceived SoA was also modulated by the position factor. The perceived SoO level varied in regard to the agent factor in that the experience of “owning” a limb was reported to be stronger in the self-agent conditions as compared to the other-agent conditions. This effect has also been observed in previous studies [28], [30], [38]. As the visual, proprioceptive and tactile feedback was very similar in the self-agent and other-agent conditions, the effect can probably not be easily attributed just to differences in sensory input per se. The only obvious difference that existed between the conditions was the presence or absence of self-agency. Hence, it is possible that the effect relates to voluntary action and its phenomenal experience. This interpretation concurs with traditional phenomenological accounts [39], more recent enactivist approaches [40]–[43] and neurocognitive theories [44]–[46] that stress the importance of bodily actions for the constitution of bodily awareness and recognition. From a neurocognitive perspective, one simple potential explanation would be that efference copy mechanisms (see introduction) may play a role not only for the recognition of an initiated action, but also for the sensory processing involved in this action [45], [47]. However, it should be noted that some studies did not find stronger SoO ratings under active than passive movements conditions [47], [48], or even found the reported SoO to be higher under passive than under active movement conditions [49]. Kalckert and Ehrsson (2014) for instance compared the RHI's inducibility under exclusively visuotactile stimulation, active movements, and passive movements, but did not find any differences in RHI strength between the three conditions. The authors concluded from this null finding that the RHI might be not only phenomenally similar, but also equally strong under all three tested RHI induction types. Additionally some neuroimaging evidence exists for rather separate neuronal networks being involved in the SoA and SoO [30]. The exact functional role of bodily actions for bodily awareness and recognition therefore remains a matter of ongoing research (see Jeannerod, 2014, or Kalckert and Ehrsson, 2014, for a more in-depth discussion of this topic).

Further, in accordance with Kalckert and Ehrsson [28] the perceived SoA level was modulated by the position factor in that the SoA level was higher in congruent and lower in incongruent conditions. Note that the only difference between these conditions was whether the artificial hand could be incorporated into the participant's body schema or not. It thus seems that the SoA becomes more vivid when it is directed towards movements of body parts perceived as ours rather than when it relates to external, disembodied objects. From a phenomenological perspective, this finding confirms the privileged role of bodily actions: *“The body is at the centre of physical action. Even when one's action ranges beyond the boundaries of one's body, as it often does, one is (typically) acting with one's body in some way”* [50]. Accordingly embodiment may modulate the experience of agency.

The incompleteness of the double dissociation between the SoA and SoO is also supported by the correlation analysis. Clear associations were not only found for the congruent self-agent and incongruent other-agent condition, but also for the congruent other-agent and incongruent self-agent condition. If the SoA and SoO were independent from each other, one would only have expected high correlations for the former two conditions, reflecting concomitant presence or absence of SoA and SoO, but not for the latter two.

The second research question addressed was whether intentional binding occurs when an artificial hand is used instead of a real hand, and if so, how intentional binding is expressed in the different experimental conditions. Except for the incongruent other-agent condition, strong binding effects that differed significantly from the neutral control condition were found. Please however note that these comparisons with the no-agency control condition should be taken with some caution, as the sensory stimulation in this condition was slightly different (interval estimation between two sounds, instead of interval estimation between a button press and a sound), and thus the observed strong binding effects could potentially also be due to some other binding mechanism (e.g. multisensory processing) unrelated to agency and intentionality.

Nevertheless, our results indicate that intentional binding is not limited to the use of one's own real hand but can also be induced by the use of an artificial hand. The known variability in results, where in some cases observed voluntary actions elicited intentional binding [16]–[18], while in others it did not [15], is also reflected in the present study. Whereas intentional binding was present in the congruent other-agent condition, intentional binding in the incongruent other-agent conditions, if present at all, was much weaker. Further research is necessary to address this issue in more detail. Interestingly, the degree of intentional binding did not vary in response to the position factor. Congruency of the artificial hand thus does not seem to be particularly important for the occurrence of intentional binding. This finding is contrary to the SoA questionnaire results where the perceived SoA level also depended on the position factor. Why hand positioning only affected explicit SoA evaluation but not intentional binding remains unclear, but may reflect that the concept of SoA was addressed differently on explicit and implicit levels. Whereas the intentional binding measure solely reflected the perceived time interval between button press and subsequent sound, the SoA questionnaire covered the experience of the finger movement itself. Hence, whereas embodiment may not be that crucial for pure action-onset/action-outcome registrations, it certainly contributes to the rich experience of action control.

Another interesting aspect of the intentional binding investigation was the influence of the agent factor. We found a trend (p = .056) indicating that intentional binding was stronger in self-agent compared to other-agent conditions. While this effect awaits independent confirmation before firm conclusions can be drawn, we speculate that it reflects some aspect of the difference in the processing of self-generated versus observed voluntary actions. This interpretation would corroborate Engbert et al.'s [15], [19] findings of stronger intentional binding in self-generated than observed voluntary actions, but would be in contrast to the previous evidence that reported no difference between the two action types [16]–[18]. Notably, the observed trend seemed to be mainly driven by differences in the two incongruent, that is disembodied, action conditions. Therefore it could be speculated that intentional binding is composed of some general causal inference mechanism and some minor self-specific aspect and that this self-specific aspect of binding more strongly comes into play in disembodied situations where it is commonly harder to infer one's own contribution into an action.

The third research question investigated the association between implicit and explicit SoA levels. Overall, the two relevant measures were only weakly correlated. In our experiment, the major difference between implicit and explicit SoA assessment was that, whereas time estimations were made immediately after each trial and thus did not involve any form of explicit agency reflection, the perceived SoA level was only assessed once after each block and therefore strongly relied on reflection, retrospection and long-term memory usage. To be sure, the present work did not allow direct testing of dissociability between the two SoA levels. However, the poor association found is consistent with the idea of an implicit SoA level that primarily reflects pre-reflective, non-conceptual monitoring of ongoing agency and an explicit SoA level that transcends the immediate situational context and implies reflective and postdictive aspects, such as long-term memory processes and contextual cues [23]. There are potentially other reasons for the poor association found between the SoA measures. In addition to the hierarchical SoA levels, the two SoA measures also targeted different aspects of agency. Whereas the intentional binding measure reflects the time experienced between an action and its sensory outcome, the SoA questionnaire reflects those SoA aspects relating to the action performance itself, and not to what is accomplished by the action. Hence, whereas intentional binding captures intentional aspects of agency, the SoA questionnaire focusses more on experiences of motor control (cf. Gallagher, 2007). A direction of future work may be the development of a questionnaire also tackling the experienced relationship between action and action outcome.

Finally, we also investigated the relationship between the perceived SoO level and strength of the proprioceptive drift. The weak associations found are incompatible with former studies [5], [28], [29], [47] that actually found moderate to strong associations in similar RHI settings. Interestingly, other studies have reported only weak associations (see e.g. Carruthers, 2013 for a discussion), or even dissociations between the two measures. Additionally functionally distinct brain areas have been reported for the SoO and for proprioceptive drift [51]. Moreover, several theoretical accounts exist that argue in favour of at least two distinct components contributing to bodily self-consciousness [52]–[55]. One refers to the “experience of owning a body”, or body ownership, as measured by the SoO questionnaire, and the other refers to the “experience of being a body with a given location within the environment”, or self-location, as measured by the proprioceptive drift [55]. Although we normally perceive our bodily self in a unique and coherent way, the phenomenal unity of self-consciousness may not be monolithic [56]. We may for example subjectively identify ourselves with the artificial hand, but still perceive the position of the embodied artificial hand at the position of our real hand. The present work did not allow body ownership to be dissociated from self-location. Nevertheless, several studies have reported links between the two measures suggesting at least some degree or overlap in these two concepts. Therefore whether our two SoO measures indeed reflect two (partially) distinct concepts, or whether the apparent discrepancies in reported results relate more to differences in operationalization or other methodology, remains unclear. While body ownership could not be explicitly dissociated from self-location, the reported SoO levels and the measured degrees of proprioceptive drifts were unrelated Hence, our results appear plausible and consistent with the idea of separate or distinct facets of bodily self-consciousness.

## Conclusions

This study demonstrates the feasibility of combining the intentional binding paradigm with the RHI. A vertical RHI-version was adapted in which the artificial hand's index finger could move and was used for executing purposeful button presses. The results provide further evidence for a partial double dissociation between the SoA and SoO, for empirically distinct and non-reducible SoA levels and for differences of intentional-binding processing between self-generated and observed voluntary actions.

## Supporting Information

Datafile S1 Individual raw data and scripts for preprocessing.
(ZIP)
