# Supplementary material for: Interplay of Agency and Ownership: The Intentional Binding and Rubber Hand Illusion Paradigm Combined
Source: PLoS One. 2014 Nov 4;9(11):e111967. doi: 10.1371/journal.pone.0111967 (PMC4219820; doi:10.1371/journal.pone.0111967)
Supplement: Datafile S1 — Individual raw data and scripts for preprocessing. (ZIP) [file pone.0111967.s001.zip › ReadMe.docx]

# Folder logfiles

- This folder contains the intentional binding logfiles for each participant.
- For each Subj there are five logfiles, four for the four main experimental conditions, one for the no-agency control condition. Please notice that for SUBJ 25, the logfile for the incongruent-self condition was corrupt and deleted.
- Each row represents one trial of the intentional binding phase, the first column the actual time interval of the respecting trial, the second column the estimated time interval of the respecting trial.
- Participant excluded from further analysis were:
  - SUBJ 11: Did not properly follow task-instructions.
  - SUBJ 25: corrupt IB data file.
  - SUBJ 24: sticking key

# major_analysis.mat

- This Matlab file automatically reads in the individual raw data files, runs through all pre-processing steps necessary to conduct all major statistical analyses and finally creates an SPSS-readable data file containing all relevant data (Questionnaire, Proprioceptive Drift and Intentional Binding).
- Participant excluded from further analysis were:
  - SUBJ 11: Did not properly follow task-instructions.
  - SUBJ 25: corrupt IB data file.
  - SUBJ 24: sticking key.

# export2spss.m

- Help function to export the preprocessed data from Matlab to SPSS (called by major_analysis.m)
- Participant excluded from further analysis:
  - SUBJ 11: Did not properly follow task-instructions.
  - SUBJ 25: Corrupt IB data file.
  - SUBJ 24: Sticking key.

# all.sav

- This SPSS-datasheet contains all the relevant preprocessed data for the statistical analyses.
- Participant excluded from further analysis:
  - SUBJ 11: Did not properly follow task-instructions.
  - SUBJ 25: Corrupt IB data file.
  - SUBJ 24: Sticking key.
